# Recent Advances in Pancreatic Cancer and Biliary Tract Cancers: Biology, Biomarkers, and Evolving Systemic Therapy

**DOI:** 10.3390/ijms27104413

**Published:** 2026-05-15

**Authors:** Ehab Takrori, Mahmoud Abdulmajid, Deepthi Devagudi, Ramsha Sohail, Zaynah Sadiq, Chris Berneau, Andrew Shenouda, Rakesh Adelli, Supriya Peshin, Sakshi Singal

**Affiliations:** 1 College of Medicine, Alfaisal University, Riyadh 11533, Saudi Arabia; etakrori@gmail.com; 2 School of Medicine, University of Jordan, Amman 11942, Jordan; abdelmajeedmahmood@gmail.com; 3 Department of Hospital Medicine, Adventist Health, Hanford, CA 93230, USA; devagudideepthi@gmail.com; 4 Department of Internal Medicine, Norton Community Hospital, Norton, VA 24273, USA; ramshas376@gmail.com (R.S.); cberneau1@gmail.com (C.B.);; 5 Washington Health, Fremont, CA 94538, USA; zaynahsadiq@gmail.com; 6 Center for Applied Research and Evaluation in Women’s Health, Department of Health Services Management & Policy, East Tennessee State University, Johnson City, TN 37614, USA; adelli@etsu.edu; 7 Department of Hematology & Oncology, East Tennessee State University, Johnson City, TN 37604, USA

**Keywords:** pancreatic ductal adenocarcinoma, biliary tract cancers, cholangiocarcinoma, gallbladder cancer, molecular profiling, targeted therapy, immunotherapy, antibody–drug conjugates, tumor microenvironment, precision oncology, treatment sequencing, biomarkers

## Abstract

Pancreatic ductal adenocarcinoma (PDAC) and biliary tract cancers (BTCs) remain highly lethal gastrointestinal malignancies because of late presentation, marked molecular heterogeneity, and limited durable benefit from conventional systemic therapy. This narrative review summarizes recent advances in both diseases, focusing on practice-informing clinical trials, biomarker-driven treatment strategies, and translational insights into tumor biology and resistance. In PDAC, progress includes refinement of perioperative management, broader germline and somatic testing, recognition of DNA damage repair-deficient subsets, and development of KRAS-directed therapies and rational combination strategies. In BTCs, especially intrahepatic cholangiocarcinoma, comprehensive molecular profiling has expanded precision oncology through actionable alterations such as *FGFR2* rearrangements, *IDH1* mutations, *HER2* amplification/overexpression, *BRAF* V600E, *NTRK* fusions, and MSI-high/dMMR status. Immunotherapy has a clearer role in selected BTC populations, whereas in PDAC benefit remains largely restricted to rare biomarker-defined subsets. Across both diseases, circulating tumor DNA is emerging as a promising tool for prognostication, minimal residual disease assessment, response monitoring, and early resistance detection. Contemporary care increasingly depends on early molecular profiling, individualized treatment sequencing, and integration of targeted therapies, biomarker-guided immunotherapy, and clinical trials.

## 1. Introduction

Pancreatic ductal adenocarcinoma (PDAC) and biliary tract cancers (BTCs), including intrahepatic and extrahepatic cholangiocarcinoma and gallbladder carcinoma, remain among the most clinically challenging gastrointestinal malignancies, largely because most patients present with advanced disease and experience high rates of recurrence even after potentially curative therapy.

In PDAC, the last decade has clarified that meaningful improvements in outcomes will require progress on multiple fronts including earlier detection and prevention, optimization of perioperative strategies, and systemic therapies that can address profound biologic heterogeneity and an immunosuppressive tumor microenvironment (TME). A recent state-of-the-field synthesis highlights how advances in early diagnosis platforms, risk stratification, and precision-treatment development are beginning to reshape PDAC care, while also underscoring persistent gaps in durable disease control and population-level implementation [1]. In parallel, BTCs have undergone a particularly visible transition into the precision oncology era. Molecular profiling is now central to contemporary BTC management because a substantial proportion of tumors, especially intrahepatic cholangiocarcinoma, harbor actionable alterations that can inform therapy selection and clinical trial matching [2].

Multidisciplinary guidance emphasizes that routine, comprehensive molecular testing (with careful attention to specimen adequacy and test selection) is increasingly necessary to identify therapeutically relevant drivers such as *FGFR2* rearrangements and *IDH1* mutations, as well as tumor-agnostic or less common targets including HER2 alterations, *BRAF* V600E, *NTRK* fusions, and MSI-high/dMMR status [2].

Recent practice-changing clinical trial data also support a broader reframing of standard therapy, particularly in advanced BTC. Building on the historical gemcitabine–cisplatin backbone, randomized phase III trials have demonstrated improved outcomes with the addition of immune checkpoint blockade in the first-line setting (e.g., durvalumab plus gemcitabine–cisplatin in TOPAZ-1 and pembrolizumab plus gemcitabine–cisplatin in KEYNOTE-966), establishing chemo-immunotherapy as a key contemporary benchmark in many settings [3]. These advances are complemented by an expanding targeted-therapy armamentarium supported by regulatory approvals for biomarker-defined subsets, including FGFR inhibitors such as pemigatinib and futibatinib for FGFR2-rearranged cholangiocarcinoma [4].

In PDAC, progress has been more incremental but increasingly biomarker informed. Intensified perioperative chemotherapy has improved outcomes after resection (e.g., adjuvant modified FOLFIRINOX), reinforcing the importance of treatment sequencing and systemic control even in ostensibly localized disease [5]. At the same time, identification of molecularly defined subgroups has enabled targeted approaches, most notably PARP inhibitor maintenance in germline *BRCA*-mutated metastatic PDAC, supporting broader adoption of germline testing and tumor profiling as part of routine care pathways [6]. Nevertheless, immunotherapy benefit in PDAC remains largely confined to rare biomarker-defined populations, motivating ongoing efforts to overcome stromal barriers and myeloid-driven immune suppression through rational combinations, as summarized in contemporary reviews of emerging PDAC strategies and immunotherapy lessons across GI malignancies [7].

**Figure 1:**
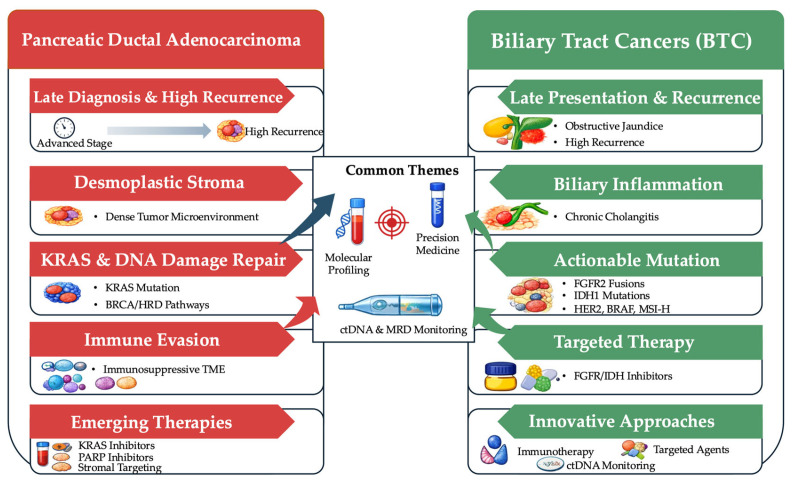
Integrated biological and therapeutic landscape of PDAC and BTC. This original schematic summarizes key disease-specific features of PDAC and BTC and highlights shared themes, including molecular profiling, precision medicine, and ctDNA/MRD monitoring. PDAC is characterized by late diagnosis, dense desmoplastic stroma, frequent *KRAS* alterations, immune evasion, and emerging therapeutic approaches, whereas BTC is characterized by biliary inflammation, actionable alterations such as *FGFR2* fusions and *IDH1* mutations, and expanding targeted and immune-based strategies. This figure was created by the authors and was not reproduced or adapted from a previously published source.

Across both PDAC and BTCs, there is growing interest in circulating tumor DNA (ctDNA) as a tool for prognostication, response assessment, and early detection of resistance. Serial ctDNA kinetics have shown promise in reflecting real-time therapeutic response in advanced pancreatic cancer, and systematic syntheses of ctDNA dynamics in metastatic and locally advanced PDAC highlight the potential role of longitudinal liquid biopsy in future risk-adapted management and trial design [8]. Finally, as therapeutic complexity increases, attention to implementation, treatment-related toxicity (including gastrointestinal immune-related adverse events), equitable access to biomarker testing, and disparities in outcomes, remains essential to ensure that advances translate into real-world benefit [9]. Figure 1 provides an integrated overview of the shared and disease-specific biological and therapeutic features of PDAC and BTC. The key clinical and molecular distinctions between the two malignancies are summarized in Table 1.

Although PDAC and BTC are both highly lethal hepato-pancreatobiliary malignancies that are often diagnosed at advanced stages and require multidisciplinary management, they differ in several clinically important ways. PDAC is characterized by a more uniform KRAS-driven biology, a dense desmoplastic and immunosuppressive microenvironment, and relatively limited targetability outside small biomarker-defined subsets, which has made treatment advances more dependent on chemotherapy optimization and selective biomarker-guided strategies [5,6]. In contrast, BTC is molecularly more heterogeneous and, particularly in intrahepatic cholangiocarcinoma, more frequently harbors actionable alterations such as *FGFR2* rearrangements and *IDH1* mutations, enabling a broader precision oncology approach alongside emerging first-line chemo-immunotherapy standards [2,3,4]. At the same time, both diseases increasingly rely on early molecular profiling, rational treatment sequencing, and evolving biomarker tools such as ctDNA to refine prognostication, response assessment, and therapeutic decision-making [6,8].

In this narrative review, we provide an integrated overview of PDAC and BTC that goes beyond parallel description of two gastrointestinal malignancies and instead emphasizes their points of convergence and divergence across molecular classification, biomarker testing, ctDNA applications, and evolving systemic treatment strategies [1,2,3,6,8]. To preserve clinical clarity while avoiding unnecessary repetition, the review uses a hybrid structure: disease-specific distinctions are retained where diagnostic pathways, surgical considerations, biomarker distributions, and therapeutic standards differ substantially, whereas shared themes are synthesized comparatively in sections addressing molecular testing, targeted therapy, immunotherapy, ctDNA/MRD monitoring, special clinical scenarios, and future directions. The novelty of this review lies in its side-by-side synthesis of how these diseases differ in targetability, immune responsiveness, resistance patterns, and clinical implementation challenges, while also highlighting shared translational priorities such as earlier molecular profiling, adaptive treatment sequencing, and biomarker-guided monitoring. In addition to summarizing recent advances, we critically examine the limitations of pivotal studies, including biomarker-restricted populations, crossover effects, single-arm designs, and gaps in real-world generalizability [3,4,6].

Our literature search followed a structured approach to identify the most relevant contemporary literature. We considered publications indexed in PubMed/MEDLINE, as well as studies published in major peer-reviewed oncology, gastroenterology, and general medical journals. We prioritized recent high-impact clinical trials, landmark randomized studies, practice-informing guideline and consensus documents, and key translational studies relevant to molecular classification, biomarker testing, ctDNA, and systemic therapy in PDAC and BTC. Emphasis was placed on studies published within the last 5–10 years, while older landmark studies were included when necessary for historical and therapeutic context. Because this article was designed as a narrative review rather than a systematic review, formal predefined inclusion and exclusion criteria were not applied. Instead, article selection was guided by clinical relevance, practice-changing potential, scientific quality, and the ability to inform an integrated discussion of evolving management strategies across both diseases.

## 2. PDAC Overview

### 2.1. Incidence and Lethality

PDAC remains a leading cause of cancer mortality worldwide and is characterized by a persistently poor long-term prognosis. Global datasets consistently place pancreatic cancer among the top causes of cancer-related death, reflecting both a rising incidence in many regions and limited curative opportunities due to late-stage presentation [10]. Contemporary syntheses further emphasize that even as systemic therapy has improved, the survival gains at a population level remain modest, underscoring the importance of earlier detection, prevention, and more effective systemic control [1,10].

The lethality of PDAC is driven by several converging factors: a biologic propensity for early dissemination, limited symptomatology until advanced stages, and a TME dominated by dense desmoplastic stroma and immunosuppressive myeloid populations that impair both drug delivery and antitumor immunity [11]. As a result, a substantial fraction of patients are diagnosed with unresectable locally advanced or metastatic disease at presentation, and recurrence remains common even after curative-intent surgery.

### 2.2. Typical Clinical Pathways of Care in PDAC

Modern management is organized around anatomic stage and resectability (resectable, borderline resectable, locally advanced unresectable, and metastatic), with treatment increasingly delivered through multidisciplinary pathways. Localized disease (resectable/borderline resectable) is surgically resectable and this remains the only potentially curative option, typically combined with systemic therapy to address micrometastatic disease risk. In fit patients, adjuvant modified FOLFIRINOX demonstrated superior survival compared with gemcitabine and has become a benchmark regimen following resection in appropriate candidates [5]. Many centers also increasingly employ neoadjuvant strategies, particularly for borderline resectable disease, to improve R0 resection rates, select for favorable tumor biology, and ensure early delivery of systemic therapy (an approach aligned with the broader systemic-first philosophy in PDAC) [11]. Locally advanced unresectable and metastatic PDAC are generally managed with systemic therapy selected according to performance status, comorbidity burden, and molecular features, with individualized consideration of local consolidation or biomarker-guided approaches in selected patients [11]. Detailed treatment sequencing is discussed in later sections and summarized in Table 1.

### 2.3. Precision Oncology Subset and Biomarker-Driven Therapy

A critical recent advancement is the recognition that germline and somatic testing can identify actionable vulnerabilities in a subset of PDAC, particularly in DNA damage repair pathways. In patients with metastatic PDAC and germline *BRCA* mutations whose disease has not progressed on first-line platinum-based chemotherapy, maintenance olaparib improved progression-free survival versus placebo (POLO), supporting routine germline testing and biomarker-informed maintenance strategies in eligible patients [6]. While broader targeted therapy success in PDAC remains limited compared with some other GI cancers, ongoing efforts focus on KRAS-directed agents, rational combinations, and microenvironment-modulating strategies designed to overcome immune exclusion [11,46].

## 3. BTC Overview

### 3.1. Incidence, Heterogeneity, and Prognosis

BTCs comprise a biologically and anatomically heterogeneous group that includes intrahepatic cholangiocarcinoma (iCCA), extrahepatic cholangiocarcinoma (eCCA), and gallbladder cancer (GBC) (with ampullary cancers often discussed alongside but frequently managed within distinct paradigms). BTCs are less common than PDAC but are associated with poor outcomes overall, with prognosis driven by stage at diagnosis, feasibility of complete surgical resection, and tumor subtype. Global data indicate that BTC incidence is increasing in multiple regions, particularly for iCCA, and risk-factor profiles vary geographically, including chronic biliary inflammation, hepatobiliary disease, and regional infectious exposures [2,25].

iCCA arises within the liver parenchyma and is notable for a comparatively high frequency of actionable genomic alterations, making comprehensive molecular profiling central to modern management [2]. eCCA arises in the biliary tree outside the liver and often presents with obstructive symptoms, yet diagnosis and curative therapy are frequently delayed; even after resection, recurrence risk remains substantial.

Gallbladder cancer is frequently aggressive and may be discovered incidentally during cholecystectomy, but many patients present with advanced disease [25]. Outcomes depend strongly on stage and resectability, and systemic therapy is typically extrapolated from broader BTC evidence where subtype-specific data are limited. Ampullary cancers are rare and biologically distinct; although curable when localized (typically via pancreaticoduodenectomy), advanced cases often require systemic therapy guided by histologic subtype and molecular features [2,25].

### 3.2. Typical Clinical Pathways of Care in BTC

For resectable BTC, surgery remains the only curative option and is typically followed by adjuvant systemic therapy in selected patients because recurrence risk remains high [19]. In advanced disease, first-line therapy has evolved from gemcitabine/cisplatin alone to chemo-immunotherapy-based standards, while second line and biomarker-guided options are increasingly shaped by molecular profiling [3,28]. These treatment pathways are discussed in greater detail in later sections and summarized in Table 1.

### 3.3. Biomarker-Driven Therapy as a Defining BTC Advance

BTCs, especially iCCA, represent one of the clearest GI oncology success stories for precision medicine, with routine molecular profiling recommended to identify actionable alterations and guide therapy selection and trial referral [2]. This paradigm includes recurrent drivers (e.g., *FGFR2* fusions/rearrangements, *IDH1* mutations) and tumor-agnostic or less frequent alterations (e.g., MSI-H/dMMR, *NTRK* fusions, *BRAF* V600E, HER2 alterations), enabling targeted therapy strategies and individualized sequencing.

## 4. Molecular Landscape and Biomarker Testing

### 4.1. PDAC: Key Genomic and Biologic Themes

Although both PDAC and BTC increasingly require molecular characterization, their biomarker landscapes differ substantially. PDAC remains dominated by KRAS-driven biology with actionable subsets concentrated in a minority of patients, whereas BTC, particularly iCCA, has a broader and more clinically mature precision oncology framework. The genomic architecture of PDAC is dominated by a relatively conserved set of recurrent driver alterations that shape tumor biology, prognosis, and therapeutic resistance. Activating *KRAS* mutations are present in approximately 85–90% of PDAC, constitutively engaging downstream pathways such as MAPK and PI3K-AKT, and are closely linked to aggressive growth, metabolic rewiring, and treatment resistance [59]. Co-alterations in core tumor suppressors, particularly *TP53*, *CDKN2A*, and *SMAD4*, are frequent and contribute to genomic instability, altered cell-cycle control, metastatic behavior, and therapy resistance, with co-mutation patterns increasingly recognized as prognostically informative [60]. In PDAC, sequencing is not used as a stand-alone diagnostic test; diagnosis remains based on clinical evaluation, imaging, and histopathologic confirmation. Rather, sequencing is used after diagnosis or when tissue is available to refine molecular classification, identify actionable alterations, guide treatment selection, support clinical trial referral, and detect resistance mechanisms [6,11,32,59,60]. Commonly assessed genes include *KRAS*, *BRCA1/2*, *PALB2*, *TP53*, *CDKN2A*, and *SMAD4*. Additional less common but clinically relevant alterations include *BRAF*, *ERBB2*, *NTRK*, *ALK*, and *NRG1*. In this section and in Table 2, we summarize biomarkers that support diagnostic refinement, molecular classification, treatment selection, risk stratification, and clinical trial referral. These include histopathologic and serum-based markers, germline and somatic molecular alterations, and tumor-specific actionable biomarkers that increasingly guide individualized care in PDAC and BTC.

While *KRAS*-mutant PDAC constitutes the majority, a clinically meaningful minority of *KRAS*-wild-type PDAC is enriched for potentially actionable alterations, including *BRAF* alterations, *ERBB2* (HER2) amplification, MSI-H/dMMR, and oncogenic fusions (e.g., *ALK*, *NTRK*, *NRG1*) [32]. This subgroup underscores why comprehensive tumor profiling can be valuable even in a cancer long perceived as target-poor, particularly when aligning patients to clinical trials or tumor-agnostic approvals.

A major actionable biologic subset in PDAC involves DNA damage repair deficiency, most notably germline *BRCA1/2* and related homologous recombination repair alterations. Germline *BRCA1/2* pathogenic variants are identified in a minority of PDAC patients, commonly reported in approximately 4–7% of cases [11,59]. Because *KRAS* mutations are highly prevalent in PDAC, many tumors with germline *BRCA1/2* alterations may still harbor concurrent *KRAS* mutations; however, the exact combined *BRCA*/*KRAS* frequency varies across cohorts and is not usually reported as a fixed clinical subgroup percentage [11,32,59]. The POLO trial established maintenance olaparib as a biomarker-directed strategy in metastatic PDAC with germline *BRCA* mutations whose disease has not progressed after platinum-based chemotherapy, supporting routine identification of this subgroup [6].

Beyond DNA sequence alterations, PDAC biology is strongly shaped by epigenetic dysregulation, including promoter hypermethylation of tumor-suppressor genes, global DNA hypomethylation, altered histone acetylation and methylation, chromatin-remodeling abnormalities, and dysregulated non-coding RNA programs such as microRNAs and long non-coding RNAs. These changes can influence tumor initiation, epithelial–mesenchymal transition, differentiation state, therapeutic plasticity, immune interactions, and resistance to therapy [61].

### 4.2. BTC: Key Actionable Alterations

BTCs are molecularly heterogeneous and, compared with PDAC, more frequently harbor clinically actionable genomic alterations, particularly in intrahepatic cholangiocarcinoma (iCCA), making upfront comprehensive molecular profiling central to modern BTC management. Rather than relying on a uniform treatment paradigm, BTC increasingly requires genomically stratified care in which biomarker identification directly informs therapeutic selection, sequencing, and trial eligibility [2,4]. The major actionable biomarkers across PDAC and BTC, including recommended testing modalities, current clinical applicability, and matched therapeutic options, are summarized in Table 2. In BTC, sequencing is similarly used to refine molecular classification and guide treatment rather than a stand-alone diagnostic test. Commonly assessed alterations include *FGFR2* fusions/rearrangements, *IDH1* mutations, *ERBB2* (HER2) amplification/overexpression, *BRAF* V600E, *NTRK* fusions, MSI/MMR status, TMB, and broader DNA damage repair alterations. Tissue NGS is commonly used for broad profiling, while RNA-based testing is particularly important for fusion detection, especially for *FGFR2* fusions in iCCA [2,31].

Among these alterations, *FGFR2* fusions/rearrangements and *IDH1* mutations are especially practice-shaping in iCCA, while additional actionable subsets include *BRAF* V600E, *ERBB2* (HER2) alterations, *NTRK* fusions, and MSI-H/dMMR [31]. A clear example of biomarker-driven treatment selection is the FDA accelerated approval of pemigatinib for previously treated, unresectable locally advanced or metastatic cholangiocarcinoma harboring FGFR2 fusions/rearrangements, based on the FIGHT-202 study [4].

**Figure 2:**
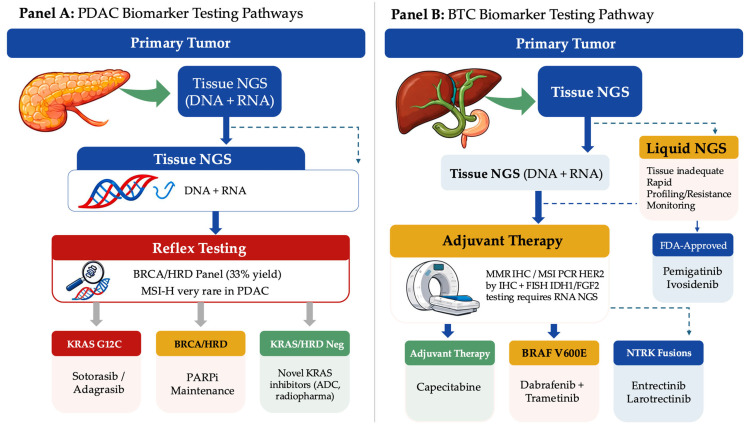
Biomarker testing pathways in PDAC and BTC. This original schematic summarizes tissue-based and liquid-biopsy approaches for identifying clinically actionable biomarkers and guiding biomarker-directed therapy in pancreatic ductal adenocarcinoma and biliary tract cancers.

By contrast, extrahepatic cholangiocarcinoma (eCCA) and gallbladder cancer show partially overlapping but distinct enrichment patterns, including relatively higher proportions of *KRAS*-pathway alterations in some cohorts and a clinically relevant frequency of HER2 amplification/overexpression, particularly in gallbladder cancer, supporting HER2-directed approaches in selected patients [12,15]. A practical biomarker testing workflow and the corresponding actionable therapeutic outputs for both PDAC and BTC are summarized in Figure 2.

## 5. Advances in Localized Disease Management

### 5.1. PDAC (Resectable, Borderline Resectable, Locally Advanced)

**Figure 3:**
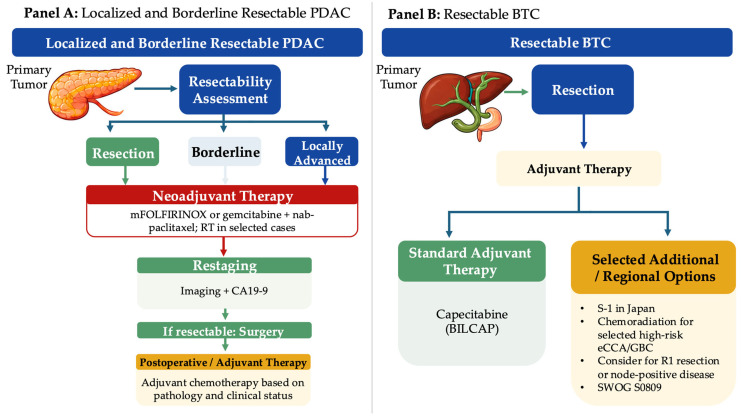
Treatment pathways for localized/borderline resectable PDAC and resectable BTC. This original schematic summarizes key management steps for localized and borderline resectable pancreatic ductal adenocarcinoma and resectable biliary tract cancers, including resectability assessment, neoadjuvant therapy, restaging, surgery when feasible, adjuvant therapy, and selected regional or risk-adapted postoperative options. Abbreviations: BTC, biliary tract cancers; CA19-9, carbohydrate antigen 19-9; eCCA, extrahepatic cholangiocarcinoma; GBC, gallbladder cancer; PDAC, pancreatic ductal adenocarcinoma; RT, radiotherapy.

Localized PDAC should be approached as a systemic disease with a local component, given the high risk of occult micrometastatic spread and the substantial recurrence rates even after apparently curative resection [1,5]. Consequently, management has evolved toward multimodal, stage- and biology-adapted care that integrates systemic therapy early, emphasizes treatment delivery at high-volume centers, and increasingly uses neoadjuvant strategies to improve patient selection and margin-negative resection rates [11]. The stage-based management pathways for localized PDAC and resectable BTC, including key decision points for neoadjuvant therapy, restaging, resection, and adjuvant treatment are summarized in Figure 3.

### 5.2. Resectable PDAC

For patients with resectable tumors and adequate physiologic reserve, surgical resection remains essential and is selected according to tumor location and anatomic involvement. Pancreaticoduodenectomy is generally used for tumors of the pancreatic head, uncinate process, or periampullary region; distal pancreatectomy, often with splenectomy, is used for tumors of the pancreatic body or tail; and total pancreatectomy is reserved for selected cases with multifocal disease, diffuse gland involvement, or anatomy that precludes a more limited oncologic resection [11,16,17,18]. In borderline resectable disease, surgery is typically considered after neoadjuvant therapy and restaging, particularly when margin-negative resection appears feasible and there is no evidence of disease progression [11,16,17,18]. Modern adjuvant standards were shaped by PRODIGE 24/CCTG PA.6, in which modified FOLFIRINOX improved survival compared with gemcitabine in resected PDAC, establishing it as a benchmark regimen for eligible patients [5].

Not all patients can tolerate mFOLFIRINOX; therefore, alternative adjuvant options remain relevant. The ESPAC-4 trial demonstrated improved overall survival with gemcitabine plus capecitabine compared with gemcitabine alone, supporting GemCap as a commonly used option in patients unsuitable for more intensive therapy [15].

### 5.3. Borderline Resectable PDAC

Borderline resectable PDAC is now commonly managed with neoadjuvant therapy to increase the probability of R0 resection, treat micrometastatic disease earlier, and identify patients with rapidly progressive biology who are unlikely to benefit from upfront surgery. This approach aligns with NCCN-endorsed strategies that recommend neoadjuvant therapy in borderline resectable disease and consider it in selected high-risk resectable cases [16].

With respect to regimen selection, the Alliance A021501 randomized phase II trial supports neoadjuvant modified FOLFIRINOX as a reference regimen in borderline resectable PDAC; notably, the arm incorporating hypofractionated radiotherapy did not outperform chemotherapy alone in that study, reinforcing ongoing uncertainty about the routine role and optimal sequencing of radiation in this setting [17]. In parallel, PREOPANC provides long-term randomized evidence for a neoadjuvant strategy using gemcitabine-based chemoradiotherapy versus upfront surgery, supporting the broader principle of preoperative treatment while highlighting that outcomes vary by regimen, patient selection, and trial design [18].

Survival outcomes after neoadjuvant therapy vary by disease stage, regimen, resectability, response to treatment, and whether patients ultimately undergo margin-negative resection. In borderline resectable PDAC, neoadjuvant modified FOLFIRINOX in Alliance A021501 was associated with favorable survival outcomes in the chemotherapy-alone arm, with 18-month overall survival reported at approximately two-thirds of patients. In PREOPANC, gemcitabine-based neoadjuvant chemoradiotherapy produced more modest median overall survival differences compared with upfront surgery but improved several surgical and long-term outcome measures, including margin-negative resection and long-term survival in selected analyses [17,18]. Therefore, the main neoadjuvant approaches discussed in this review include modified FOLFIRINOX and gemcitabine-based chemoradiotherapy, with radiotherapy considered selectively rather than uniformly [16,17,18].

### 5.4. Locally Advanced (Unresectable) PDAC

Locally advanced PDAC remains challenging because durable local control is difficult and systemic progression is common. Current practice generally begins with induction multi-agent chemotherapy (regimen choice guided by performance status and comorbidities), followed by reassessment for conversion to resection in highly selected responders or consideration of consolidative local therapy for symptom control and local progression risk [11,16,17,18].

The role of consolidative chemoradiation remains debated. In LAP07, among patients with locally advanced disease controlled after initial chemotherapy, subsequent chemoradiotherapy improved local control but did not improve overall survival compared with continued chemotherapy, underscoring why radiation is typically individualized rather than uniformly applied [62]. Importantly, the modern development focus in locally advanced PDAC is shifting toward optimizing systemic induction, defining conversion criteria, and exploring biology-driven combinations (including microenvironment-modulating strategies) rather than relying solely on local intensification [11,62].

When radiation-based approaches are compared with chemotherapy-focused strategies, the available evidence does not support a single universally superior approach for all patients with locally advanced or borderline resectable PDAC. In LAP07, consolidative chemoradiotherapy improved local control but did not improve overall survival compared with continued chemotherapy, whereas neoadjuvant modified FOLFIRINOX-based strategies have become favored in many fit patients because of stronger systemic disease control and favorable outcomes in selected borderline resectable cohorts [17,18,62]. Toxicity profiles also differ. Chemotherapy-based approaches are more often limited by systemic adverse effects such as cytopenias, fatigue, gastrointestinal toxicity, and neuropathy, whereas radiation-based approaches may add local gastrointestinal toxicity, fatigue, nausea, duodenal or gastric irritation, and biliary or hepatic considerations depending on treatment field. Therefore, the best approach is individualized rather than universal, with selection guided by resectability, performance status, comorbidities, baseline nutritional status, tumor-vessel anatomy, response to induction therapy, local symptom burden, and multidisciplinary assessment [16,17,18,62].

### 5.5. BTC (Resectable Disease)

For resectable BTC, including iCCA, eCCA, and gallbladder cancer, complete surgical resection with negative margins remains the central curative modality, with the operative approach determined by anatomic subtype, extent of biliary or hepatic involvement, vascular involvement, and nodal disease [12,13]. For intrahepatic cholangiocarcinoma, surgery typically involves partial hepatectomy or major hepatectomy with regional lymphadenectomy when technically feasible. For perihilar or extrahepatic cholangiocarcinoma, resection may require bile duct resection with hepatic resection, caudate lobectomy, lymphadenectomy, and biliary reconstruction depending on tumor location and longitudinal ductal involvement. For gallbladder cancer, simple cholecystectomy may be adequate only for very early incidental disease, whereas more advanced resectable tumors generally require extended cholecystectomy with hepatic wedge or segment IVb/V resection and regional lymphadenectomy [12,13]. However, recurrence after surgery is common, making adjuvant therapy a key component of contemporary management [13,19,20,21,22].

### 5.6. Adjuvant Systemic Therapy

The BILCAP phase III trial established adjuvant capecitabine as the most widely adopted reference standard following curative-intent resection of BTC, and this recommendation is reflected in major guidelines and consensus statements [19]. Gemcitabine and capecitabine are both cytotoxic antimetabolites but differ in formulation, mechanism, and common clinical use. Gemcitabine is an intravenously administered nucleoside analogue incorporated into DNA and commonly used in pancreatic and biliary chemotherapy combinations, whereas capecitabine is an oral prodrug of 5-fluorouracil that inhibits thymidylate synthase and is commonly used as adjuvant therapy after resected BTC [19]. It is worth noting that adjuvant strategies are not fully uniform across regions. In Japan, the randomized phase III JCOG1202 (ASCOT) trial demonstrated a survival benefit with adjuvant S-1 compared with observation after resection of BTC, supporting geographic variation when S-1 is routinely available and used [20]. Although gemcitabine–cisplatin is a foundational regimen in advanced BTC, it is not the established adjuvant standard; the phase III ACTICCA-1 study was designed to compare adjuvant GemCis with capecitabine, and guideline documents have highlighted that its results are awaited to further refine adjuvant selection [21].

### 5.7. Adjuvant Chemoradiation in Selected BTC

For eCCA and gallbladder cancer, particularly in patients at high risk of locoregional relapse or with margin-positive disease, adjuvant chemoradiation is sometimes considered. SWOG S0809 provides prospective phase II data supporting a structured approach using systemic chemotherapy followed by concurrent chemoradiation in resected eCCA/gallbladder cancer, and it remains an important evidence base informing real-world practice where randomized data are limited [22].

## 6. Advances in Metastatic Systemic Therapy

### 6.1. PDAC: Chemotherapy Backbones and Sequencing

The contrast between PDAC and BTC is particularly evident in metastatic systemic therapy. PDAC treatment remains largely chemotherapy-based with selective biomarker-driven maintenance or trial-based targeted approaches, whereas BTC has more rapidly incorporated first-line chemo-immunotherapy and later-line molecularly matched therapies [3,5,6]. Metastatic PDAC remains a high-mortality disease, but systemic therapy has evolved toward risk-adapted, multi-agent treatment strategies with increasing attention to sequencing, toxicity management, and selective maintenance approaches [24,27]. For fit patients (typically ECOG 0-1), the principal first-line chemotherapy backbones are FOLFIRINOX and gemcitabine plus nab-paclitaxel, with regimen choice guided by performance status, comorbidity profile, anticipated toxicities, and patient goals [24]. Key practice-changing metastatic PDAC trials and their therapeutic implications are summarized in Table 3.

FOLFIRINOX became a modern first-line benchmark after the phase III PRODIGE 4/ACCORD 11 trial demonstrated improved survival compared with gemcitabine, albeit with greater toxicity requiring careful patient selection and supportive care [5]. Gemcitabine plus nab-paclitaxel, supported by the phase III MPACT trial, remains an alternative evidence-based first-line option, particularly when clinicians seek a combination regimen that may be more suitable for selected patients based on tolerability considerations [24]. In these pivotal first-line metastatic PDAC trials, median overall survival was 11.1 months with FOLFIRINOX versus 6.8 months with gemcitabine in PRODIGE 4/ACCORD 11, and 8.5 months with gemcitabine plus nab-paclitaxel versus 6.7 months with gemcitabine alone in MPACT [5,24].

The toxicity profile of FOLFIRINOX is clinically important when selecting and sequencing therapy. Short-term adverse effects commonly include fatigue, nausea, vomiting, diarrhea, mucositis, anorexia, dehydration, cytopenias, neutropenia or febrile neutropenia, and acute cholinergic symptoms related to irinotecan. Medium-term toxicities may include cumulative fatigue, weight loss, nutritional decline, recurrent cytopenias, infection risk, chemotherapy delays, and progressive sensory neuropathy, particularly related to oxaliplatin exposure. Longer-term or persistent toxicities may include chronic peripheral neuropathy, functional decline, sarcopenia, and reduced tolerance of later-line therapy, making early dose adjustment and supportive care essential in selected patients [5,11]. Treatment individualization is currently based on performance status, age and physiologic reserve, baseline neuropathy, nutritional status or sarcopenia, renal and hepatic function, bilirubin level or biliary obstruction, comorbidity burden, patient goals, and anticipated tolerance of cumulative toxicity [11]. Emerging and actively evaluated factors include molecular predictors of platinum sensitivity, particularly homologous recombination repair deficiency and germline or somatic *BRCA1/2* or related DDR alterations, as well as dynamic biomarkers such as CA19-9 and ctDNA kinetics that may help refine response assessment, treatment adaptation, and clinical trial selection [6]. DNA synthesis inhibiting cytotoxic agents are used in PDAC and BTC because these tumors often have rapid proliferative activity and remain largely dependent on cytotoxic chemotherapy backbones in the absence of broadly targetable alterations; these agents impair DNA replication or damage repair, leading to tumor-cell death [5,11,24].

Platinum agents, including oxaliplatin in FOLFIRINOX and cisplatin in BTC regimens, exert antitumor activity primarily by forming intra- and inter-strand DNA crosslinks, thereby impairing DNA replication and transcription and promoting apoptosis in rapidly dividing tumor cells. This mechanism is clinically relevant in tumors with impaired DNA damage repair or homologous recombination repair, including selected *BRCA1/2*- or *PALB2*-altered cancers [6,11]. Clinically important toxicities vary by agent but include fatigue, nausea and vomiting, cytopenias, hypersensitivity reactions, and peripheral neuropathy. Oxaliplatin is particularly associated with acute cold-induced dysesthesia and cumulative sensory neuropathy, whereas cisplatin is more strongly associated with nephrotoxicity, ototoxicity, and electrolyte wasting. These toxicity profiles support individualized regimen selection, dose modification, hydration and electrolyte monitoring, and early recognition of cumulative neurotoxicity or organ dysfunction [5,11,24].

Gemcitabine-specific toxicities also influence regimen selection and dose intensity. Although generally less intensive than FOLFIRINOX, gemcitabine can cause myelosuppression, particularly neutropenia, anemia, and thrombocytopenia, as well as fatigue, nausea, rash, fever or flu-like symptoms, and transient liver enzyme elevations. Rare but clinically important toxicities include pneumonitis or interstitial lung disease, thrombotic microangiopathy, capillary leak syndrome, and radiation recall; these events require prompt recognition, treatment interruption, and supportive management when suspected [3,11,24].

Supportive medications used during chemotherapy are selected according to regimen-specific toxicity risk and patient factors [5,11,24]. Common adjuncts include 5-HT3 receptor antagonists, dexamethasone, NK1 receptor antagonists, and olanzapine for antiemetic prophylaxis; atropine for acute irinotecan-related cholinergic symptoms; loperamide or diphenoxylate-atropine for chemotherapy-associated diarrhea; granulocyte colony-stimulating factor in patients at increased risk of febrile neutropenia or after neutropenic complications; analgesics and neuropathy-directed agents for chemotherapy-induced neuropathy; and hydration, electrolyte replacement, and renal-protective measures during cisplatin-containing therapy. These interventions are individualized based on baseline organ function, prior toxicity, nutritional status, infection risk, and treatment intent [5,11,24].

Second-line therapy is largely shaped by prior treatment exposure. After progression on gemcitabine-based therapy, the NAPOLI-1 trial established nanoliposomal irinotecan (nal-IRI) plus 5-FU/leucovorin as a key evidence-supported option, with median overall survival of 6.1 months versus 4.2 months with 5-FU/leucovorin alone [27]. After progression on FOLFIRINOX, many patients transition to a gemcitabine-based regimen if clinically appropriate, while trial enrollment remains strongly encouraged whenever feasible [24,27].

**Figure 4:**
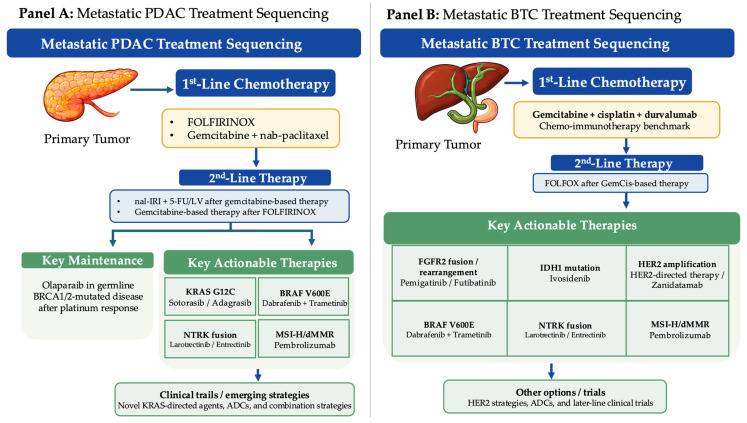
Treatment sequencing maps for metastatic PDAC and BTC. This original schematic summarizes common first-line regimens, selected maintenance and second-line options, biomarker-directed therapies, and later-line clinical trial considerations for metastatic pancreatic ductal adenocarcinoma and biliary tract cancers. Abbreviations: ADC, antibody–drug conjugate; BTC, biliary tract cancers; GemCis, gemcitabine plus cisplatin; MSI-H/dMMR, microsatellite instability-high/deficient mismatch repair; nal-IRI, nanoliposomal irinotecan; PDAC, pancreatic ductal adenocarcinoma.

An additional sequencing advance is the incorporation of biomarker-driven maintenance in selected patients. In metastatic PDAC with germline *BRCA* mutations whose disease has not progressed after platinum-based induction, maintenance olaparib improved progression-free survival in the POLO trial, supporting routine germline testing and a distinct maintenance strategy for this subgroup [6]. An overview of contemporary treatment sequencing for metastatic PDAC and BTC, including chemotherapy backbones, biomarker-defined options, and later-line pathways, is summarized in Figure 4.

### 6.2. BTC: First-Line and Second-Line Evolution

The metastatic BTC landscape has changed rapidly over the last few years, driven by chemo-immunotherapy adoption in the first line and systematic integration of molecularly targeted therapies for actionable subgroups, especially in iCCA. Historically, gemcitabine/cisplatin was the standard backbone. More recently, the phase III TOPAZ-1 trial evaluated durvalumab, an anti-PD-L1 immune checkpoint inhibitor that blocks PD-L1-mediated immune suppression and enhances antitumor T-cell activity, in combination with gemcitabine/cisplatin in advanced BTC. TOPAZ-1 demonstrated an overall survival benefit with durvalumab plus gemcitabine/cisplatin versus chemotherapy alone, with median overall survival of 12.8 months versus 11.5 months, and longer follow-up has shown that this benefit can persist over time [3]. In parallel, KEYNOTE-966 reported a statistically significant overall survival improvement with pembrolizumab plus gemcitabine/cisplatin, with median overall survival of 12.7 months versus 10.9 months, supporting chemo-immunotherapy as an increasingly common first-line standard in appropriately selected patients [26]. After progression on first-line gemcitabine/cisplatin-based therapy, the ABC-06 randomized trial established FOLFOX plus active symptom control as a reference second-line option, demonstrating a clinically meaningful survival improvement with median overall survival of 6.2 months versus 5.3 months compared with active symptom control alone [28].

A single fixed percentage of BTC patients who develop chemoresistance is difficult to define because trials typically report response rate, progression-free survival, and disease control rather than chemoresistance as a separate endpoint. Clinically, chemoresistance is most often reflected by progression after gemcitabine/cisplatin-based therapy; despite improvements with first-line chemo-immunotherapy, median progression-free survival in pivotal advanced BTC trials remains limited, indicating that most patients ultimately require second-line therapy, molecularly matched treatment, or clinical trial enrollment [3,25,26,28].

BTC is now one of the most target-rich GI malignancies, making early comprehensive profiling essential. For FGFR2 fusions/rearrangements, pemigatinib has an established role in previously treated cholangiocarcinoma, expanding targeted options for this biomarker-defined subgroup [4]. For *IDH1*-mutated cholangiocarcinoma, ClarIDHy demonstrated clinical benefit with ivosidenib versus placebo (with crossover considerations), supporting *IDH1* as a clinically actionable driver [36]. Finally, MSI-H/dMMR BTC, although uncommon, represents a tumor-agnostic immunotherapy-responsive subgroup where PD-1 blockade (e.g., pembrolizumab) is an established strategy, consistent with broader GI immunotherapy principles summarized in your review sources [46]. The major metastatic treatment benchmarks and biomarker-defined systemic therapy options are summarized in Table 3.

## 7. Targeted Therapy

### 7.1. PDAC Targeted Therapy

Targeted therapy in PDAC remains constrained by dominant KRAS-driven biology, dense stromal and immune barriers, and the relatively low prevalence of broadly actionable alterations. Nevertheless, meaningful progress has been made in biomarker-defined subsets, where targeted strategies now function either as standard options for selected patients or as emerging trial-based approaches [43]. The principal biomarker-defined targeted strategies, their typical clinical placement, and key resistance considerations are summarized in Table 4.

The most clinically established targeted paradigm in PDAC is the exploitation of homologous recombination repair deficiency, particularly germline *BRCA1/2* alterations. In the phase III POLO trial, maintenance olaparib prolonged progression-free survival compared with placebo in patients with germline *BRCA*-mutated metastatic PDAC whose disease had not progressed after platinum-based chemotherapy, supporting biomarker-guided maintenance therapy and reinforcing the importance of routine germline testing [6].

Current KRAS-directed strategies in PDAC include allele-specific inhibitors, with the most clinically advanced data involving tumors harboring alterations that encode the p.Gly12Cys protein alteration, commonly abbreviated as KRAS p.G12C. KRAS undergoes alternative splicing to generate the KRAS4A and KRAS4B isoforms; however, codon 12 lies within the coding region shared by both isoforms, and clinical studies of KRAS-directed agents generally classify tumors by the encoded protein alteration rather than by isoform-specific expression. Although KRAS p.G12C is uncommon in PDAC, early phase studies of sotorasib and adagrasib have demonstrated objective responses and disease control in previously treated pancreatic cancer, providing proof of concept for direct KRAS inhibition in selected patients [44,45]. Because KRAS p.G12C represents only a small minority of pancreatic cancers and acquired resistance remains a major challenge, development is expanding toward broader KRAS-targeted strategies, including approaches directed against more common protein alterations such as p.G12D and p.G12V, downstream MAPK-pathway combinations, and resistance-delaying strategies [1,11,43,44,45].

Additional actionable opportunities are enriched in *KRAS*-wild-type PDAC, including *BRAF* alterations, *ERBB2* (HER2) amplification, MSI-H/dMMR, and oncogenic fusions such as *NTRK*, *ALK*, and *NRG1*, which may enable tumor-agnostic therapy or trial enrollment in selected cases [63]. At the same time, mutation-specific KRAS inhibitors and rational pathway-combination strategies are advancing, but for most PDAC patients these approaches remain investigational rather than established standard therapy.

Efforts to target the TME have also been central to PDAC drug development. However, broad stromal depletion strategies such as hedgehog pathway inhibition have not translated into meaningful clinical benefit; for example, adding vismodegib to chemotherapy did not improve outcomes, underscoring the limitations of simply reducing stromal content without adequately addressing immune suppression or adaptive escape [64]. Current approaches therefore increasingly focus on stromal and immune reprogramming rather than nonspecific depletion.

Overall, the clearest standard-of-care targeted application in PDAC remains biomarker-guided therapy for DDR/HRD-defined subsets. In contrast, KRAS-directed therapy represents a rapidly advancing but still largely investigational frontier, with current efforts focused on improving durability, overcoming resistance, and extending direct targeting beyond rare KRAS p.G12C-altered tumors [43,44,45].

### 7.2. BTC Targeted Therapy

BTC, particularly iCCA, has become one of the clearest gastrointestinal examples of clinically meaningful precision oncology because actionable alterations are comparatively common and directly affect treatment sequencing. As a result, comprehensive molecular profiling is now a prerequisite for modern BTC management rather than an optional adjunct [2,12]. The principal biomarker-defined targeted therapy strategies, their clinical placement, and major resistance considerations are summarized in Table 4.

Among these, *FGFR2* alterations define a major actionable subset in iCCA. The FDA granted accelerated approval to pemigatinib for previously treated, unresectable locally advanced or metastatic cholangiocarcinoma harboring *FGFR2* fusions/rearrangements, based on the FIGHT-202 study, establishing FGFR inhibition as a cornerstone biomarker-guided strategy in this population [4]. Futibatinib has further expanded this space; it is an irreversible covalent FGFR1-4 inhibitor that blocks aberrant *FGFR* signaling driven by *FGFR2* fusions/rearrangements, with activity in *FGFR2*-rearranged iCCA and potential relevance in some acquired resistance settings [37].

For *IDH1*-mutant cholangiocarcinoma, ivosidenib demonstrated clinical benefit in the phase III ClarIDHy trial, further reinforcing *IDH1* as a clinically actionable driver and supporting early testing for this alteration in advanced disease [36,38]. HER2 amplification, particularly relevant in gallbladder cancer and a subset of cholangiocarcinomas, has also become increasingly actionable, with zanidatamab showing meaningful activity in HER2-positive metastatic BTC and strengthening the role of HER2-directed therapy in selected patients [39].

Additional but less common actionable subsets include *BRAF* V600E, where dabrafenib plus trametinib demonstrated activity in the ROAR basket trial, as well as *NTRK* fusions and MSI-H/dMMR, which may permit tumor-agnostic targeted or immune-based strategies when identified [40]. Taken together, BTC targeted therapy is best understood not as a single treatment advance, but as a sequencing framework driven by upfront profiling, molecular subtype, and resistance patterns.

Despite these advances, several limitations temper interpretation of the BTC targeted-therapy literature and help explain why real-world application can lag behind trial efficacy. Much of the strongest evidence outside first-line chemo-immunotherapy derives from biomarker-selected, single-arm studies, which are highly informative for proof of activity but less definitive for comparative effectiveness, optimal sequencing, and durability across broader clinical populations [39,40]. In addition, crossover designs, such as in ClarIDHy, complicate overall survival interpretation, while acquired on-target and bypass resistance remain major challenges after initially effective targeted therapy, particularly in *FGFR2*-rearranged disease [36,37]. More broadly, real-world benefit depends not only on drug activity but also on access to high-quality tissue acquisition, comprehensive molecular testing, repeat profiling at progression, and referral pathways capable of translating genomic findings into treatment decisions or clinical trial enrollment [2,12,31].

## 8. Immunotherapy and Immune-Based Combinations

### 8.1. Why PDAC Is Immune-Cold

Immunotherapy represents one of the clearest areas of divergence between PDAC and BTC, with PDAC remaining largely immune-cold and resistant to checkpoint blockade outside rare biomarker-defined subsets, while BTC has established first-line chemoimmunotherapy as a treatment benchmark for many patients with advanced disease [3,26,46]. PDAC has been consistently characterized as an immune-cold malignancy because single-agent immune checkpoint blockade rarely produces meaningful benefit outside of uncommon biomarker-defined subsets [7,30,46]. This resistance reflects more than the general presence of an immunosuppressive TME; rather, it arises from a set of interconnected stromal, cellular, and signaling mechanisms that collectively impair antitumor immunity. A dense desmoplastic stroma, abnormal vasculature, and extensive extracellular matrix deposition restrict effective immune-cell trafficking and promote stromal exclusion of effector T cells. Cancer-associated fibroblasts (CAFs) play a central role in this process by shaping the fibrotic architecture of the tumor, modulating cytokine networks, and reinforcing a microenvironment that favors immune evasion over immune activation [7,30,46].

In parallel, PDAC is enriched with suppressive immune populations, including tumor-associated macrophages, myeloid-derived suppressor cells, and regulatory T cells, which blunt dendritic-cell function, impair cytotoxic T-cell priming, and limit effective effector responses. The main T-cell populations include CD8+ cytotoxic T cells, which mediate tumor-cell killing; CD4+ helper T cells, which coordinate immune responses; regulatory T cells, which suppress antitumor immunity; and exhausted T cells, which show reduced effector function after chronic antigen exposure [30]. Several signaling pathways further sustain this immunosuppressive state. TGF-β signaling contributes to fibroinflammatory remodeling, immune exclusion, and reduced T-cell penetration; IL-6/STAT3 signaling promotes tumor survival, myeloid polarization, and an anti-inflammatory tumor contexture; and IDO-mediated tryptophan catabolism contributes to local immune tolerance and T-cell dysfunction. In addition, most PDACs display a relatively low tumor mutational burden, resulting in fewer strongly immunogenic neoantigens and weaker baseline immune recognition [7,30,46].

These features contribute to distinct noninflamed immune phenotypes within PDAC, including immune-excluded tumors, in which immune cells are present but remain confined largely to the stromal compartment, and immune-desert tumors, in which meaningful antitumor immune infiltration is minimal or absent. Collectively, these biologic features explain why PDAC does not behave like a classic checkpoint-responsive tumor and why current immunotherapy development is focused less on PD-1/PD-L1 blockade alone and more on rational combinations designed to improve antigen presentation, remodel stromal and myeloid suppression, and enhance T-cell infiltration and effector function [30,47,48,49,50].

### 8.2. Mechanisms of Primary Immunotherapy Resistance in PDAC

Primary immunotherapy resistance in PDAC reflects more than the broad presence of an immunosuppressive TME. Multiple interrelated mechanisms contribute to immune evasion and limited checkpoint inhibitor efficacy. First, the dense desmoplastic stroma and abnormal vasculature create a physical and functional barrier to effective T-cell trafficking and intratumoral immune-cell penetration. Second, PDAC is enriched for suppressive myeloid populations, including tumor-associated macrophages and myeloid-derived suppressor cells, as well as regulatory T cells, which collectively impair cytotoxic T-cell priming and effector function. Third, many PDACs exhibit low baseline immunogenicity, with relatively low tumor mutational burden and limited neoantigenicity, reducing the likelihood of spontaneous immune recognition. Additional resistance mechanisms may include impaired antigen presentation, defective dendritic-cell activation, and adaptive compensatory signaling pathways that maintain immune exclusion even when checkpoint pathways are inhibited. Together, these features help explain why single-agent immunotherapy has shown limited benefit in unselected PDAC and why current development is increasingly focused on rational combination strategies designed to improve priming, trafficking, and microenvironmental reprogramming [7,30,46].

### 8.3. BTC Immunotherapy: Where Benefit Is Real and Who Benefits Most

Compared with PDAC, BTC has a clearer, and now practice-changing role for immunotherapy in the first-line advanced disease setting when combined with chemotherapy. The phase III TOPAZ-1 trial demonstrated an overall survival benefit for durvalumab plus gemcitabine/cisplatin versus gemcitabine/cisplatin alone, and extended follow-up confirms the benefit is sustained [3]. Similarly, KEYNOTE-966 showed an overall survival improvement with pembrolizumab plus gemcitabine/cisplatin compared with chemotherapy alone [26]. These trials support chemo-immunotherapy as a contemporary benchmark for many patients with unresectable or metastatic BTC.

Beyond chemo-immunotherapy, a subset-driven paradigm remains essential. MSI-H/dMMR (and in some cases high TMB) BTC represents a tumor-agnostic immunotherapy-responsive group where PD-1 blockade can be highly active, reinforcing the need for routine molecular profiling [26]. PD-L1 expression may enrich for response in some settings, but it is not uniformly predictive across BTC subtypes and should be interpreted in the context of the broader molecular and clinical picture.

### 8.4. Combination Strategies Under Active Development

Given PDAC’s immune resistance and the need to extend immunotherapy benefit beyond selected BTC populations, multiple immune-based combinations are advancing. The strongest rationale is to convert cold tumors into inflamed, T-cell-accessible tumors and/or to intensify antigen-specific immunity in minimal residual disease settings. The strategies highlighted below were selected as representative, non-exhaustive examples based on their mechanistic relevance to PDAC/BTC immune resistance and the availability of translational or early clinical data in these disease contexts; other emerging approaches, including STING agonists and oncolytic virus-based strategies, are also of interest but are beyond the selective scope of this section.

(1) Vaccines (neoantigen and shared-antigen approaches)

Personalized neoantigen vaccines have provided proof-of-concept that PDAC, despite low mutational burden, can generate durable neoantigen-specific T-cell responses. These vaccines use patient-specific tumor-derived neoantigens identified from individual tumor sequencing rather than a fixed shared antigen; early clinical work with individualized mRNA-lipoplex neoantigen vaccination (autogene cevumeran) in surgically resected PDAC demonstrated induction of mutation-specific T cells and suggested a link between vaccine-induced responses and delayed recurrence in responders [47]. Reported toxicities were generally consistent with immune activation and perioperative combination therapy, including transient systemic symptoms, infusion or injection-related reactions, fatigue, fever, chills, and potential immune-related events when combined with checkpoint blockade [47,48]. Ongoing studies are exploring optimal pairing with checkpoint blockade and perioperative systemic therapy [48].

(2) CD40 agonism (innate activation and improved priming)

CD40 agonists aim to activate antigen-presenting cells and enhance T-cell priming, potentially overcoming poor baseline priming in PDAC. A phase 1b study combining the CD40 agonist sotigalimab (APX005M) with gemcitabine/nab-paclitaxel, with or without nivolumab, established feasibility and generated a signal that supports continued development of CD40-based combinations [49].

(3) Myeloid and trafficking modulation (e.g., CXCR4 axis)

Because myeloid suppression and impaired trafficking are central to PDAC immune resistance, strategies targeting these pathways have become prominent. In the phase IIa COMBAT/KEYNOTE-202 trial, motixafortide/BL-8040 combined with pembrolizumab and chemotherapy showed feasibility and signs of activity in metastatic PDAC, supporting the broader concept that immune-cell trafficking modulation may sensitize PDAC to checkpoint blockade in combination settings [50].

(4) T-cell engagers and multispecific platforms

T-cell engagers (TCEs) and multispecific immune engagers are being engineered to redirect T cells toward tumor cells and may help bypass weak endogenous priming. While most clinical successes have been in hematologic malignancies, the platform is rapidly evolving for solid tumors, with active trial development addressing potency, safety, and tumor-selective activation [65]. Preclinical work in cholangiocarcinoma also supports potential synergy between chemotherapy and PD-L1 × CD3 engager formats [66].

(5) Radiotherapy synergy

Radiotherapy can increase antigen release and modulate the TME, providing a rationale for combination with checkpoint blockade. Contemporary reviews emphasize that the key question is not whether synergy exists biologically, but which dose/fractionation, sequencing, and patient subsets can translate that biology into clinical benefit, particularly in PDAC where systemic failure remains common [67].

Despite strong biologic rationale, many immune-based combination strategies in PDAC have not yet translated into durable or reproducible clinical benefit. One major reason is that PDAC immune resistance is not driven by a single dominant barrier but by multiple overlapping mechanisms, including stromal exclusion, CAF-mediated remodeling, myeloid-dominant suppression, poor antigen priming, and adaptive compensatory signaling [30]. As a result, interventions that target only one component of this network often produce biologic activity without sufficient clinical impact. In addition, promising preclinical findings have frequently been difficult to reproduce in patients because of tumor heterogeneity, limited biomarker-based patient selection, uncertainty regarding optimal sequencing and combination partners, and the possibility that strategies such as broad stromal modulation may disrupt tumor architecture without restoring effective antitumor immunity, underscoring why progress has remained incremental and why future success will likely depend on more precisely selected, resistance-aware combination approaches [30,49,50,64,67].

## 9. Emerging Therapeutic Platforms Beyond Immune-Combination Strategies

Beyond the immune-combination strategies discussed above, additional progress in PDAC and BTC is being driven by emerging therapeutic platforms designed to improve tumor selectivity, broaden treatment options, and address resistance mechanisms. The most clinically relevant examples at present include antibody–drug conjugates (ADCs), HER2-directed bispecific platforms, T-cell engagers, multispecific immune engagers, and device-based approaches, particularly where human clinical data or strong translational rationale are available [66,68,69]. Selected emerging modalities with the most clinically relevant human data in PDAC and BTC are summarized in Table 5.

### 9.1. Antibody–Drug Conjugates

ADCs combine a tumor-targeting antibody with a cytotoxic payload, allowing selective delivery of potent therapy while seeking to improve the therapeutic index. In PDAC, CLDN18.2 has emerged as a relevant surface target, and early phase clinical data for the CLDN18.2-targeting ADC IBI343 have shown encouraging activity signals in advanced solid tumors, including PDAC, supporting continued development and biomarker-enriched selection strategies [68]. In BTC, ADC development is also advancing around targetable surface proteins, particularly HER2 in selected cholangiocarcinoma and gallbladder cancer subsets. Across both diseases, the major challenges remain heterogeneous antigen expression, on-target/off-tumor toxicity, and determination of optimal sequencing and combination partners [39,68].

### 9.2. Bispecific Antibodies, T-Cell Engagers, and Multispecific Platforms

Bispecific and multispecific antibody formats aim either to redirect immune effector cells toward tumor targets or to intensify pathway inhibition through dual binding. For T-cell-redirecting platforms, the intended mechanism is to bring tumor cells into close proximity with cytotoxic T cells, most often through CD3 engagement, thereby promoting immune synapse formation, T-cell activation, cytokine release, and tumor-cell killing; however, efficacy in PDAC and BTC remains investigational, with limitations related to cytokine release syndrome, tumor selectivity, immune-cell trafficking, and stromal barriers in solid tumors [65,66]. The antibody-based agents discussed in this review primarily target immune checkpoints, innate immune activation pathways, or tumor-associated antigens, including PD-1/PD-L1, CD40, HER2, CLDN18.2, and T-cell redirection targets such as PD-L1 × CD3 [65,68]. In BTC, zanidatamab represents one of the clearest clinically relevant advances among bispecific antibody platforms, although its mechanism is HER2-directed dual-epitope binding rather than T-cell redirection. By binding two distinct HER2 extracellular epitopes, zanidatamab promotes dual HER2 blockade, receptor internalization, and immune-mediated antitumor activity. Published human trial data in HER2-positive BTC demonstrated clinically meaningful response activity with a generally manageable safety profile, with toxicities most commonly including diarrhea and infusion-related reactions [39]. More broadly, T-cell engagers and related multispecific platforms remain promising but are still earlier in development, particularly in stromal-rich tumors such as PDAC, where safety, trafficking, and target-selection challenges remain important barriers to wider clinical application [39,65,66,69].

### 9.3. Where the Field Is Going

At present, the most clinically mature emerging modalities in PDAC and BTC are those supported by published human trial data and biomarker-guided patient selection. Across these platforms, future progress will likely depend on clearer target-definition thresholds, improved toxicity management, and rational integration with existing chemotherapy, immunotherapy, and targeted-therapy backbones [69]. In parallel, immune-priming strategies such as individualized neoantigen vaccination remain an important investigational direction in PDAC, with early data supporting durable vaccine-induced T-cell responses and ongoing trials evaluating integration with checkpoint blockade and perioperative systemic therapy [70,71]. More speculative approaches remain important from a translational standpoint but will require stronger clinical validation before their place in routine care can be defined.

## 10. ctDNA, MRD, and Response Monitoring

### 10.1. ctDNA for Prognosis and Early Relapse in Localized Disease (MRD)

In both PDAC and BTC, ctDNA and MRD strategies are being evaluated as tools for prognostication, relapse detection, response monitoring, and resistance tracking; however, they remain outside routine standard clinical practice in most settings and are currently used primarily in investigational, translational, or research-directed contexts. In localized PDAC and resected BTC, relapse after curative-intent therapy is frequently driven by occult micrometastatic disease. ctDNA-based MRD assays, tumor-informed (personalized) or tumor-agnostic, are being evaluated to detect residual disease at a molecular level before radiographic recurrence becomes evident [51,54,55]. In PDAC, aggregated evidence indicates that postoperative ctDNA positivity stratifies patients into a high-recurrence-risk group and can precede radiographic relapse by weeks to months in some cohorts, supporting ctDNA as a promising tool for postoperative risk stratification and clinical trial enrichment [8,51].

BTC is moving in a similar direction. Tumor-informed ctDNA MRD detection has been shown to be feasible in resected biliary tract cancer and appears to correlate with recurrence risk, a clinically important signal given the limitations of current clinicopathologic risk models [54]. Longitudinal ctDNA monitoring has also been evaluated in resected cholangiocarcinoma cohorts, with ctDNA dynamics correlating with relapse risk and potentially informing postoperative surveillance strategies [55].

**Figure 5:**
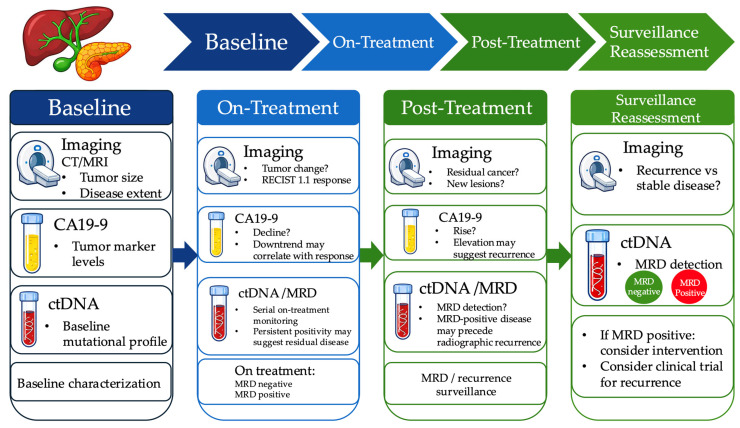
Longitudinal integration of imaging, CA19-9, and ctDNA/MRD assessment in PDAC and BTC. This original schematic summarizes complementary monitoring approaches at baseline, during treatment, after treatment, and during surveillance or reassessment. It highlights the combined use of cross-sectional imaging, CA19-9 trends, and ctDNA/MRD status for response assessment, recurrence detection, and risk-adapted follow-up. Abbreviations: CA19-9, carbohydrate antigen 19-9; ctDNA, circulating tumor DNA; MRD, minimal residual disease; RECIST 1.1, Response Evaluation Criteria in Solid Tumors version 1.1.

At present, the most clinically supported role of ctDNA/MRD in localized PDAC and BTC is prognostic risk stratification, including identification of patients at high risk of recurrence and earlier molecular detection of relapse relative to imaging in some cohorts [51,54,55]. By contrast, interventional applications such as treatment escalation in MRD-positive patients, de-escalation in persistently ctDNA-negative patients, or adjuvant treatment selection based directly on ctDNA status remain investigational and should currently be viewed as research-directed strategies rather than routine standard-of-care [51]. A practical framework integrating ctDNA/MRD with cross-sectional imaging and CA19-9 for longitudinal response assessment and surveillance in PDAC and BTC is shown in Figure 5.

### 10.2. ctDNA for Treatment Response and Resistance in Metastatic and Locally Advanced Disease

In metastatic and locally advanced PDAC, ctDNA is increasingly evaluated as a real-time biomarker of tumor burden and early therapeutic efficacy, potentially offering response information earlier than conventional imaging. Early changes in ctDNA levels during systemic therapy have been associated with clinical outcomes in multiple studies, supporting the hypothesis that serial ctDNA dynamics can function as an on-treatment response biomarker and may help identify non-responders earlier in the treatment course [52].

These concepts are reinforced by broader translational syntheses showing that ctDNA can capture clonal evolution and emerging resistance mechanisms, enabling earlier molecular reassessment and potential trial matching [53]. In BTC, ctDNA profiling is similarly attractive for noninvasive identification of actionable alterations and tracking of resistance patterns, particularly under targeted therapy selection pressure [29].

ctDNA is best positioned at present as a complementary monitoring tool, particularly when tissue is limited, imaging findings are equivocal, or rapid molecular reassessment could open a targeted therapy or clinical trial option, rather than a replacement for radiographic response assessment [51]. Thus, while serial ctDNA dynamics are increasingly informative as a complementary prognostic and response-monitoring biomarker, using ctDNA alone to trigger a treatment change in routine practice remains an evolving strategy that still requires prospective validation.

### 10.3. Integration with Imaging and CA19-9

Standard monitoring in PDAC and BTC relies on cross-sectional imaging and CA19-9, but both have limitations: imaging may lag behind biologic change, and CA19-9 is confounded by cholestasis/inflammation and is non-informative in Lewis antigen-negative patients. CA19-9 remains useful as an accessible adjunct, and in some analyses, it can precede radiographic recurrence after resection [23].

ctDNA adds a third dimension, molecular disease activity, that may provide earlier evidence of progression or relapse in selected patients and can support a more adaptive monitoring strategy. Reviews synthesizing PDAC ctDNA evidence highlight that ctDNA may detect recurrence before radiographic confirmation in a subset of patients and may offer complementary value to CA19-9 and imaging, although performance varies by assay type, sampling schedule, and tumor shedding biology [51]. Motobayashi et al. further support the clinical relevance of serial ctDNA dynamics in metastatic and locally advanced PDAC as an outcomes-associated biomarker, strengthening the rationale for incorporating ctDNA into response and surveillance frameworks as evidence matures [52].

Despite this promise, important gaps remain before ctDNA can be integrated as a routine stand-alone decision tool in PDAC and BTC. Current limitations include assay heterogeneity, differences in analytic sensitivity, variable tumor shedding, inconsistent sampling schedules, and the risk of overinterpreting molecular changes without prospective interventional validation [29,51,52,53]. In addition, ctDNA performance is likely to vary by disease burden, treatment context, and anatomic site, which complicates universal application across perioperative, locally advanced, and metastatic settings. Accordingly, ctDNA is best viewed at present as a complementary biomarker that can refine risk stratification and response assessment alongside imaging and CA19-9, rather than as a replacement for established monitoring frameworks or a definitive trigger for treatment escalation or de-escalation [23,29,51,52,53].

In practical terms, baseline biomarker assessment before treatment should include CA19-9, when informative, cross-sectional imaging, and molecular profiling, including ctDNA when clinically available or research-directed [23,29,51,52,53]. During and after treatment, CA19-9 trends may stabilize or decline with response, while rising CA19-9 or re-emergent/increasing ctDNA may suggest residual disease, recurrence, or progression, although interpretation must account for biliary obstruction, inflammation, assay variability, and tumor-shedding biology. Monitoring should therefore be longitudinal and risk-adapted rather than based on a fixed universal duration; in routine practice, surveillance is generally continued throughout active therapy and during post-treatment follow-up, with intensity guided by disease stage, treatment intent, symptoms, imaging findings, and biomarker reliability [23,51].

## 11. Special Clinical Scenarios

### 11.1. Older Adults and Frail Patients: Balancing Efficacy with Tolerability

A growing proportion of PDAC and BTC patients are older and/or have limited physiologic reserve, making regimen choice and dosing strategy central to outcomes. In metastatic PDAC, modified-dose FOLFIRINOX has been used in carefully selected older adults, with retrospective data suggesting preserved efficacy and manageable toxicity when dose adjustments and supportive care are applied appropriately [1,11,56]. These data support an individualized approach in which treatment selection incorporates performance status, baseline neuropathy risk, nutritional status or sarcopenia, renal function, and patient goals. In this context, fit older patients may still derive benefit from multi-agent therapy, whereas others may be better served by less intensive regimens, earlier palliative co-management, and closer toxicity surveillance [56]. Early reassessment after treatment initiation is also supported by the need to identify intolerance, functional decline, or emerging complications before cumulative toxicity becomes treatment-limiting [11,56].

### 11.2. Cholestasis, Biliary Obstruction, and Hepatic Dysfunction (BTC-Heavy, but Relevant Across Both)

Biliary obstruction is common in BTC and can also occur in PDAC involving the pancreatic head, complicating systemic therapy through increased infection risk, treatment delay, and altered hepatic drug handling [11,14]. Guideline-based and multidisciplinary management therefore emphasizes effective biliary drainage when feasible, careful timing of chemotherapy around interventions, and monitoring for stent-related complications [14]. A key real-world issue is chemotherapy delivery in the setting of elevated bilirubin, because pivotal first-line trials establishing gemcitabine/cisplatin excluded patients above certain bilirubin thresholds. Nonetheless, dedicated analyses suggest that cisplatin/gemcitabine can be administered cautiously in selected patients with persistent jaundice after drainage and stabilization, supporting an individualized approach to dose selection and escalation as hepatic function improves [57].

### 11.3. CA19-9 Non-Secretors and Biomarker Interpretation Pitfalls

CA19-9 is a sialylated Lewis blood group antigen used clinically as a supportive serum biomarker for disease burden, treatment response, and recurrence monitoring in PDAC and BTC, although it is not sufficiently specific or sensitive to diagnose cancer by itself. In apparently healthy individuals, CA19-9 is generally low, with the commonly used upper limit of normal around 37 U/mL, but levels may rise in benign biliary obstruction, cholangitis, pancreatitis, and other inflammatory conditions. CA19-9 is elevated in many patients with PDAC, particularly in advanced disease, but approximately 5–10% of individuals are Lewis antigen-negative and may have absent or low CA19-9 secretion, limiting its reliability for surveillance and response assessment [14,23,72]. In BTC, interpretation is further complicated by the fact that cholestasis and inflammation may elevate CA19-9 independently of tumor burden. These limitations support greater reliance on cross-sectional imaging and, where available, complementary biomarkers such as ctDNA in cases where CA19-9 appears discordant with the clinical picture. Accordingly, persistently low or unchanged CA19-9 despite evident disease progression should raise the possibility of non-secretion or biomarker confounding, particularly in the setting of biliary obstruction, cholangitis, or recent instrumentation [14,72].

### 11.4. Margin-Positive/High-Risk Resected BTC: When to Consider Chemoradiation

Evidence for adjuvant chemoradiation in resected BTC remains less robust than in several other gastrointestinal malignancies, but it is still considered in selected high-risk settings, particularly after R1 resection or in node-positive disease involving eCCA or gallbladder cancer. The prospective phase II SWOG S0809 study supports a structured strategy of systemic chemotherapy followed by concurrent chemoradiation and remains an important reference point in multidisciplinary discussions and guideline-informed practice [22].

### 11.5. Germline Testing and Family Implications (Especially PDAC)

Because actionable DDR alterations can shape therapy (e.g., platinum sensitivity and PARP inhibitor maintenance pathways) and because PDAC is one of the cancers where universal germline testing is widely recommended in modern practice, germline results carry both therapeutic and familial implications. Incorporating genetic counseling, cascade testing where appropriate, and clear documentation of results in the oncology workflow can improve both care and prevention opportunities in relatives [1,6,11].

### 11.6. Delivery-Limiting Toxicities and Supportive Care That Affect Sequencing

In PDAC and BTC, mortality usually reflects progressive metastatic or locally advanced disease, cancer-related cachexia, organ dysfunction, infection or biliary complications, thromboembolic events, and treatment-related complications [1,11,14,57]. In both PDAC and BTC, the ability to remain on effective therapy is often determined by how well predictable toxicities are anticipated and managed over time. Cumulative neuropathy from oxaliplatin- or taxane-based regimens can influence later-line treatment options and may prompt dose modification, de-escalation, or regimen change when function becomes compromised. Malnutrition and sarcopenia, especially common in PDAC, are also clinically important because they can reduce treatment tolerance and adversely affect outcomes, supporting early nutritional assessment and supportive intervention as part of treatment planning [11,56].

## 12. Future Directions

Future progress in PDAC and BTC will depend not only on expanding the therapeutic armamentarium, but also on improving how tumor biology is translated into clinically actionable care [2,11,12]. A major priority across both malignancies is the more consistent implementation of precision oncology, including timely referral for suspicious symptoms or imaging findings, rapid access to pancreas- or hepatobiliary-protocol imaging, high-quality tissue acquisition, comprehensive molecular profiling, multidisciplinary interpretation of results, and equitable delivery of guideline-concordant care across diverse patient populations [2,12,58]. At the same time, biomarker-defined and tumor-agnostic treatment strategies are likely to play an increasingly important role in treatment development and sequencing, particularly in subsets characterized by MSI-H/dMMR, *NTRK* fusions, *FGFR2* rearrangements/fusions, and HER2 amplification/overexpression, where continued refinement of patient selection and therapeutic positioning remains necessary [33,34,35,41,42]. Thus, future progress in both diseases will require not only new therapies, but also more integrated frameworks that connect baseline molecular profiling, treatment sequencing, resistance monitoring, and real-world implementation [29,51,58]. Findings from ongoing, unpublished, or preliminary clinical trials should therefore be interpreted as investigational and hypothesis-generating until supported by peer-reviewed publication, mature follow-up, and guideline incorporation.

In PDAC, future gains will likely depend on overcoming the biological constraints that have limited durable benefit from both immunotherapy and many targeted approaches, particularly stromal exclusion, myeloid-dominant immune suppression, and adaptive resistance [7,11,30]. Progress in this setting will require better biomarker-guided combination strategies, improved identification of patients most likely to benefit from immune-modulating approaches, and prospective validation of ctDNA- and MRD-informed treatment adaptation. In BTC, key priorities include optimizing treatment sequencing after chemo-immunotherapy or targeted therapy, improving management of acquired resistance, and integrating repeat molecular profiling more effectively at the time of progression [29,37,38,39,40]. In addition, regional treatment platforms such as hepatic arterial infusion pump (HAIP) therapy for unresectable, liver-confined iCCA highlight how locoregional intensification strategies may expand the therapeutic window in selected settings and may inform future gastrointestinal cancer trial design and therapeutic delivery frameworks [73,74]. More broadly, both PDAC and BTC are moving toward adaptive care models that combine baseline molecular characterization with longitudinal disease monitoring, with the long-term aim of replacing static line-based treatment paradigms with more biologically informed and response-adaptive strategies [29,58].

## 13. Conclusions

Recent advances in PDAC and BTCs are increasingly redefining care through the convergence of biomarker-driven oncology, evolving systemic therapy standards, and emerging therapeutic platforms. In PDAC, progress has been most evident in optimized perioperative and metastatic multi-agent chemotherapy approaches, alongside meaningful advances for molecularly defined subsets, particularly DNA damage repair/homologous recombination deficiency populations where platinum sensitivity and PARP inhibitor maintenance are clinically actionable, yet outcomes remain constrained by late diagnosis, early dissemination, and a stromal- and myeloid-dominant immunosuppressive microenvironment that limits immunotherapy benefit outside rare biomarker-defined contexts. In contrast, BTCs, especially intrahepatic cholangiocarcinoma, have more rapidly entered the precision oncology era, with routine comprehensive profiling enabling targeted therapy for *FGFR2* fusions/rearrangements, *IDH1* mutations, and additional actionable subsets (e.g., HER2, *BRAF* V600E, *NTRK*), while chemo-immunotherapy has shifted the first-line benchmark for many patients with advanced disease. Across both malignancies, ctDNA and MRD strategies are emerging as promising tools for prognostication, response monitoring, and early relapse detection, supported by growing evidence that serial ctDNA kinetics can correlate with outcomes in metastatic and locally advanced PDAC. Looking forward, durable survival gains will likely depend on earlier detection and interception, prospective validation of ctDNA-guided treatment algorithms, and rational combination approaches that overcome resistance and remodel the tumor microenvironment, particularly in PDAC, while continued development of ADCs, bispecific immune engagers, vaccines, and device-based therapies should be integrated into biomarker-enriched, sequencing-focused trials and implemented alongside equitable access to molecular testing and high-quality multidisciplinary care.

## Figures and Tables

**Table 1:** PDAC versus BTC: Clinical and molecular overview.

Feature	PDAC	BTC (iCCA/eCCA/GBC)	Preferred Diagnostic Method
Major subtypes	Predominantly PDAC [1,10,11]	iCCA, eCCA, GBC ± ampullary [2,12,13]	PDAC: Histopathologic confirmation from EUS-guided or image-guided biopsy [11]. BTC: Histopathologic confirmation with anatomic classification by cross-sectional imaging and pathology [12,13]
Typical presentation	Often late; nonspecific symptoms (weight loss, abdominal/back pain); pancreatic head tumors may cause jaundice [1,10,11]	Often late; eCCA frequently presents with obstructive jaundice; iCCA often presents with liver mass symptoms or incidental imaging findings [12,13,14]	PDAC: Pancreatic-protocol CT or MRI ± EUS-guided tissue sampling [11]. BTC: Cross-sectional imaging (CT/MRI) ± MRCP/ERCP/EUS-guided or percutaneous biopsy depending on site [12,13,14]
Curative-intent option	Surgical resection according to tumor location, including pancreaticoduodenectomy for head/uncinate tumors, distal pancreatectomy for body/tail tumors, or selected total pancreatectomy; combined with perioperative systemic therapy in appropriate resectable/borderline cases [5,11,15,16,17,18]	Subtype-specific resection, including hepatectomy for iCCA, bile duct/hepatic resection with reconstruction for selected eCCA/perihilar tumors, or extended cholecystectomy for resectable gallbladder cancer, followed by adjuvant therapy when appropriate [13,19,20,21,22]	PDAC: Pancreatic-protocol CT + multidisciplinary staging ± diagnostic EUS/laparoscopy in selected cases [11,16,17,18]. BTC: Cross-sectional hepatobiliary staging ± MRCP/ERCP and surgical evaluation [12,13]
Key localized-disease themes	High recurrence risk systemic therapy integral; neoadjuvant used increasingly in borderline resectable [5,11,15,16,17,18]	High recurrence risk adjuvant therapy central; chemoradiation considered in selected high-risk eCCA/GBC [13,19,20,21,22]	PDAC: Pancreatic-protocol CT, CA19-9, and multidisciplinary resectability assessment [11,16,17,18,23]. BTC: CT/MRI ± MRCP, CA19-9, and hepatobiliary surgical staging [12,13,14]
First-line metastatic benchmark	Multi-agent chemotherapy: FOLFIRINOX or gemcitabine + nab-paclitaxel [5,24]	Gemcitabine + cisplatin + PD-1/PD-L1 inhibitor (chemo-immunotherapy benchmark for many patients) [3,25,26]	PDAC: Histologic confirmation + baseline CT/MRI and performance-status assessment; molecular testing in selected or guideline-recommended contexts [6,11]. BTC: Histologic confirmation + baseline CT/MRI with routine molecular profiling in advanced disease [2,12,13]
Second-line metastatic benchmark	Depends on first line; nal-IRI + 5-FU/LV after gem-based therapy is a key evidence-supported option [27]	FOLFOX after progression on GemCis-based therapy [28]	PDAC: Restaging CT/MRI ± CA19-9 and tumor/germline molecular reassessment when relevant [6,11,23,27]. BTC: Restaging CT/MRI with comprehensive molecular profiling if not already performed and repeat profiling when feasible [2,12,28,29]
Dominant biologic barriers	Dense desmoplastic stroma; immunosuppressive myeloid-rich TME; low baseline immunogenicity (immune-cold) [7,11,30]	Marker inter-tumor heterogeneity; biliary obstruction/infection risk; mixed immunogenicity depending on subtype/biomarkers [2,12,13,14]	PDAC: Tissue histopathology + tumor molecular profiling when clinically indicated [6,11]. BTC: Histopathology plus comprehensive molecular profiling, especially in iCCA [2,12,31]
High-yield actionable alterations	DDR/HRD (BRCA1/2, PALB2); rare KRAS-wt actionable events (MSI-H/dMMR, NTRK, HER2, BRAF) [6,32,33,34,35]	iCCA enriched for FGFR2 fusions/rearrangements and IDH1 mutations; additional actionable subsets (HER2, BRAF V600E, NTRK, MSI-H/dMMR) [2,31,33,34,35,36,37,38,39,40,41,42]	PDAC: Tissue NGS + germline testing for DDR/HRR-associated subsets [6,11]. BTC: Comprehensive tissue NGS; RNA-based NGS preferred for fusion detection, especially FGFR2 in iCCA [2,12,31]
Targeted therapy impact	Standard in small biomarker-defined subsets (e.g., PARP maintenance after platinum response in BRCA); otherwise mostly trial-based [6,43,44,45]	Major clinical impact in biomarker-defined subgroups (FGFR2, IDH1, HER2, BRAF V600E; tumor-agnostic MSI-H/NTRK) [4,33,34,35,36,37,38,39,40,41,42]	PDAC: Molecular profiling for selected actionable subsets; germline testing central for BRCA-associated therapy [6,11]. BTC: Broad molecular profiling at diagnosis of advanced disease to guide biomarker-directed therapy [2,12,31]
Role of immunotherapy	Limited benefit outside rare MSI-H/dMMR; combinations under active study [7,30,33,46,47,48,49,50]	Clear benefit in first-line chemo-immunotherapy for many; high responses in MSI-H/dMMR subgroup [3,25,26,33,46]	PDAC: MMR/MSI testing for rare MSI-H/dMMR subset [33]. BTC: MMR/MSI testing ± broader molecular profiling; PD-L1 not used as a stand-alone determinant [2,3,12,25,26,33]
Role of ctDNA/MRD	Emerging for MRD detection, prognosis, response kinetics, resistance tracking; not yet universal standard [8,51,52,53]	Emerging for profiling when tissue limited, resistance tracking (esp. on targeted therapy), and MRD research [29,54,55]	PDAC: Investigational ctDNA/MRD assays alongside imaging and CA19-9 [23,51,52,53]. BTC: Investigational ctDNA/MRD assays, particularly when tissue is limited or for research monitoring [29,54,55]
Common implementation challenges	Cachexia/sarcopenia; chemo tolerance; access to high-volume surgical care; rapid progression limits trial eligibility [1,11,56]	Cholestasis/hepatic dysfunction affecting dosing; biliary drainage/stent infections; tissue adequacy for NGS and fusion detection [2,12,14,57]	PDAC: Adequate tissue acquisition, germline testing logistics, and timely multidisciplinary evaluation [6,11]. BTC: Tissue adequacy for NGS/fusion detection, biliary instrumentation effects, and repeat profiling logistics [2,12,14,31,57]
Major unmet needs	Earlier detection/interception; effective microenvironment remodeling; durable systemic control [1,7,11,30]	Improved early diagnosis; resistance management after targeted therapy; better predictive biomarkers for IO/targeted sequencing [2,12,29,37,38,39,40]	PDAC: Earlier detection tools, more sensitive molecular stratification, and validated MRD strategies [1,51,52,53]. BTC: Earlier diagnosis, resistance monitoring, and broader access to comprehensive biomarker testing [2,12,29,54,55,58]

Abbreviations: BTC, biliary tract cancers; DDR, DNA damage repair; eCCA, extrahepatic cholangiocarcinoma; GBC, gallbladder cancer; HRD, homologous recombination deficiency; iCCA, intrahepatic cholangiocarcinoma; MRD, minimal residual disease; MSI-H/dMMR, microsatellite instability-high/deficient mismatch repair; nal-IRI, nanoliposomal irinotecan; NGS, next generation sequencing; PDAC, pancreatic ductal adenocarcinoma; TME, tumor microenvironment.

**Table 2:** Actionable biomarkers in PDAC and BTC: Recommended testing modality and matched therapies.

Biomarker/Alteration	Tumor Type(s) Where Most Relevant	Preferred Test(s)	Matched Therapy/Clinical Action	Key Evidence
BRCA1/2 (±other HRR: PALB2, etc.)	PDAC; BTC (subset)	Germline testing; tumor NGS for somatic HRR	Platinum sensitivity (clinical); maintenance PARP inhibitor after response/stable disease on platinum in metastatic setting (standard in germline BRCA-mutated PDAC; selective/less established in other HRR settings)	POLO trial, olaparib maintenance [6]
HRD/DDR (tumor) (somatic BRCA/HRR genes)	PDAC; BTC	Tumor NGS (DNA) ± germline	Consider platinum-based backbone; consider clinical trial (emerging/selective application, not uniform standard of care outside defined subsets)	Modern PDAC precision oncology frameworks [1,7,11]
MSI-H/dMMR	PDAC (rare); BTC (rare but actionable)	IHC for MMR protein and/or MSI PCR; MSI via NGS panel	PD-1 inhibitor (tumor agnostic standard of care when identified)	KEYNOTE-158 non-colorectal MSI-H/dMMR cohort supporting pembrolizumab activity in MSI-H/dMMR solid tumors [33]
High TMB	PDAC (uncommon); BTC (subset)	NGS panel reporting TMB	Consider PD-1 inhibitor in appropriate tumor-agnostic contexts; not a routine broad standard in PDAC/BTC	Tumor-agnostic immunotherapy evidence summarized in KEYNOTE-158-related data [33]
NTRK fusion	PDAC (very rare); BTC (rare)	RNA-based NGS preferred for fusions (or DNA + RNA)	TRK inhibitor (tumor agnostic standard of care when identified, though rare)	Larotrectinib and entrectinib pooled tumor-agnostic studies [34,35]
FGFR2 fusion/rearrangement	iCCA	RNA NGS (best for fusions) ± DNA NGS	FGFR inhibitor after progression on first-line therapy (standard biomarker-guided option in advanced iCCA)	Pemigatinib FDA approval summary and FIGHT-202; Futibatinib FOENIX-CCA2 [4,37,41]
IDH1 mutation	iCCA	DNA NGS	Ivosidenib after prior therapy (standard biomarker-guided option in advanced IDH1-mutant iCCA)	ClarIDHy [36]
BRAF V600E	PDAC (rare); BTC (iCCA/GBC)	DNA NGS	BRAF + MEK inhibition (standard tumor-agnostic/biomarker-guided option in selected patients)	ROAR basket trial [40]
HER2 (ERBB2) amplification/overexpression	PDAC (rare KRAS-wild type subset); BTC (esp. GBC and subset of CCA)	IHC ± ISH/FISH; NGS can detect amplification	HER2-directed therapy, often after progression (increasingly practice-informing, but still evolving across BTC subtypes and lines of therapy)	BTC molecular testing HERIZON-BTC-01 zanidatamab data [2,39,42]
KRAS p.G12C	PDAC (rare); BTC (rare)	DNA NGS	Consider KRAS p.G12C inhibitor (emerging/investigational in PDAC/BTC; not routine standard)	KRAS-targeted therapy overview; sotorasib and adagrasib clinical data [1,11,44,45]
PD-L1 expression	PDAC (limited utility); BTC (context-dependent)	IHC	Context-dependent predictive biomarker; not a direct therapeutic target or stand-alone treatment-selection marker in PDAC or BTC. Should be interpreted alongside broader clinical, pathologic, and molecular context.	First-line BTC chemo-immunotherapy evidence: TOPAZ-1 and KEYNOTE-966 [3,25,26]

Abbreviations: BTC, biliary tract cancers; CCA, cholangiocarcinoma; DDR, DNA damage repair; dMMR, deficient mismatch repair; FGFR2, fibroblast growth factor receptor 2; GBC, gallbladder cancer; HRD, homologous recombination deficiency; HRR, homologous recombination repair; IHC, immunohistochemistry; ISH, in situ hybridization; iCCA, intrahepatic cholangiocarcinoma; MSI-H, microsatellite instability-high; NGS, next-generation sequencing; PDAC, pancreatic ductal adenocarcinoma; TMB, tumor mutational burden. References in the “Key evidence” column support the corresponding biomarker, preferred testing approach, and matched therapeutic or clinical action.

**Table 3:** Practice-changing systemic therapy trials in metastatic PDAC and BTC.

Diagnosis	Line/Setting	Regimen (Trial Arm)	Comparator	Key Outcome	Practice Impact	Key Evidence
PDAC	First-line metastatic	FOLFIRINOX	Gemcitabine	Improved OS vs. gemcitabine; higher toxicity	Establishes FOLFIRINOX as a first-line benchmark for ECOG 0-1	PRODIGE 4/ACCORD 11 [5]
PDAC	First-line metastatic	Gemcitabine + nab-paclitaxel	Gemcitabine	Improved OS vs. gemcitabine	Alternative first-line benchmark, often favored when FOLFIRINOX not ideal	MPACT [24]
PDAC	Second-line metastatic (after gemcitabine-based therapy)	Nal-IRI + 5-FU/LV	5-FU/LV	Improved OS vs. 5-FU/LV	Evidence-based second-line option after gem-based progression	NAPOLI-1 [27]
PDAC	Maintenance (metastatic; after platinum response)	Olaparib (BRCA mutated)	Placebo	Improved PFS	Biomarker-defined maintenance strategy; supports universal germline testing	POLO [6]
BTC	First-line unresectable/metastatic	Durvalumab + GemCis	GemCis	Improved OS vs. GemCis	Chemo-IO benchmark for many BTC patients	TOPAZ-1 [3]
BTC	First-line unresectable/metastatic	Pembrolizumab + GemCis	GemCis	Improved OS vs. GemCis	Supports PD-1-based chemo-IO as another first-line benchmark	KEYNOTE-966 [25,26]
BTC	Second-line after GemCis-based therapy	FOLFOX + active symptom control	Active symptom control	Improved OS	Evidence-based reference second-line chemotherapy	ABC-06 [28]
BTC (iCCA)	Biomarker-defined, previously treated	Ivosidenib (IDH1 mutant)	Placebo (crossover allowed)	Improved PFS; OS interpretation affected by crossover	Establishes IDH1 as actionable driver in iCCA	ClarIDHy [36]
BTC (iCCA)	Biomarker-defined, previously treated	Pemigatinib (FGFR2 fusion/rearrangement)	Single arm (no randomized comparator)	Clinically meaningful ORR in molecularly selected population	Targeted option after progression on standard therapy	FIGHT-202 and FDA approval summary [4,41]
BTC (iCCA)	Biomarker-defined, previously treated	Futibatinib (FGFR2 fusion/rearrangement)	Single arm (no randomized comparator)	Clinically meaningful ORR; activity in some resistance contexts	Next-generation FGFR option; informs resistance sequencing	FOENIX-CCA2 [37]

Abbreviations: BTC, biliary tract cancers; GemCis, gemcitabine/cisplatin; iCCA, intrahepatic cholangiocarcinoma; nal-IRI, nanoliposomal irinotecan; ORR, objective response rate; OS, overall survival; PDAC, pancreatic ductal adenocarcinoma; PFS, progression free survival. References in the “Key evidence” column support the corresponding regimen, comparator, outcome, and practice impact.

**Table 4:** Targeted therapy in PDAC and BTC: biomarker-defined strategies, typical sequencing, and resistance considerations.

Alteration/Target	Tumor Type(s)	Typical Clinical Placement	Key Agent	Resistance	Key Evidence
BRCA1/2 (HRR)	PDAC	Maintenance after platinum response/stable disease in metastatic setting (established standard of care in eligible PDAC patients)	Olaparib	Progression: switch systemic backbone; clinical trial	POLO [6]
FGFR2 fusion/rearrangement	iCCA	Usually after first-line GemCis ±immunotherapy or after progression on standard therapy (established biomarker-guided option in iCCA)	Pemigatinib; Futibatinib	Acquired resistance may occur through on-target FGFR2 kinase mutations or bypass signaling; consider switching FGFR inhibitor, trial combination, or ctDNA-guided mutation assessment	Pemigatinib FDA approval summary and FIGHT-202; futibatinib FOENIX-CCA2 [4,37,41]
IDH1 mutation	iCCA	After prior therapy, commonly post first-line (established biomarker-guided option in iCCA)	Ivosidenib	Resistance may involve pathway bypass; consider chemotherapy/IO per prior exposure or trials	ClarIDHy [36]
HER2 (ERBB2) amplification/overexpression	BTC (esp. GBC)	After progression on standard chemo/chemo-IO; earlier in trials (emerging but increasingly practice-relevant; not yet uniform standard across settings)	Zanidatamab; trastuzumab-based regimens in some settings	Resistance includes antigen heterogeneity/escape and downstream pathway activation switch HER2 strategy or enroll in HER2 trial	BTC molecular testing guidance and HERIZON-BTC-01 [2,39,42]
BRAF V600E	BTC (subset)	After prior therapy (selective biomarker-guided option; established in molecularly defined patients but uncommon)	Dabrafenib + trametinib	Resistance may occur through MAPK reactivation; consider clinical trial enrollment or alternate systemic therapy	ROAR basket [40]
NTRK fusion	PDAC (rare), BTC (rare)	Any line when identified (tumor agnostic standard of care, though rare)	Larotrectinib; Entrectinib	Resistance via on-target mutations next-gen TRK inhibitors (trial)	Larotrectinib and entrectinib pooled tumor-agnostic studies [34,35]
MSI-H/dMMR	PDAC (rare), BTC (rare)	Any line when identified (tumor agnostic standard of care)	Pembrolizumab	Primary resistance uncommon but possible; consider trials/alternate systemic therapy	KEYNOTE-158 MSI-H/dMMR cohort [33]
FGFR2 resistance monitoring via ctDNA	iCCA	During FGFR therapy at and at progression (emerging practice tool/investigational adjunct, not yet routine standard)	ctDNA profiling to detect emerging FGFR2 kinase mutations	Supports selection of next FGFR inhibitor/trial enrollment	BTC liquid biopsy review and molecular testing guidance [2,29]

Abbreviations: BTC, biliary tract cancers; CCA, cholangiocarcinoma; ctDNA, circulating tumor DNA; dMMR, deficient mismatch repair; GBC, gallbladder cancer; HRR, homologous recombination repair; iCCA, intrahepatic cholangiocarcinoma; MSI-H, microsatellite instability-high; PDAC, pancreatic ductal adenocarcinoma. References in the “Key evidence” column support the corresponding biomarker-defined strategy, clinical placement, resistance consideration, and matched therapeutic option.

**Table 5:** Selected emerging modalities in PDAC and BTC: development landscape and key considerations.

Modality	Target/Platform	Disease Relevance	Typical Development Setting	Rationale	Key Limitations/Consideration	Key Evidence
ADCs	CLDN18.2 ADC (e.g., IBI343)	PDAC and BTC	Early phase metastatic; biomarker-enriched	Delivers potent payload to antigen-positive tumor cells to improve therapeutic index	Antigen heterogeneity; off-tumor toxicity; expression cutoffs needed	CLDN18.2 ADC early clinical data [68]
Bispecific antibodies/HER2 platforms	Zanidatamab (HER2 bispecific)	BTC	Post-progression metastatic; trials moving earlier	Dual-epitope HER2 bindings may improve depth and duration of response	Requires validated HER2 testing; resistance via pathway bypass/heterogeneity	HERIZON-BTC-01 zanidatamab data [39,42]
TCEs/multispecific immune engagers	PD-L1xCD3, other TCE constructs	PDAC and BTC	Early phase; combination-focused	Redirects T cells to tumor cells, potentially bypassing poor endogenous priming	CRS risk; trafficking barrier in stroma-rich tumors; target selection critical	Solid tumor TCE development overview and preclinical cholangiocarcinoma PD-L1xCD3 data [65,66]
Device-based therapy	Tumor Treating Fields	PDAC	Phase III/practice translation	Non-pharmacologic modality added to chemo without systemic toxicity	Access, adherence/device logistics; real-world integration	PANOVA-3 [69]

Abbreviations: ADC, antibody–drug conjugate; BTC, biliary tract cancers; CRS, cytokine release syndrome; PDAC, pancreatic ductal adenocarcinoma; TCE, T-cell engager. References in the “Key evidence” column support the corresponding emerging modality, therapeutic target, clinical rationale, and key limitations.

## Data Availability

No new data were created or analyzed in this study. Data sharing is not applicable to this article.
